# Construction and In Vitro Digestibility of Recrystallized Starch Encapsulated in Calcium Alginate Beads

**DOI:** 10.3390/foods12122379

**Published:** 2023-06-15

**Authors:** Kaili Qin, Rongyu Zhang, Weili Qin, Na Ji, Yang Qin, Lei Dai, Liu Xiong, Qingjie Sun

**Affiliations:** 1 College of Food Science and Engineering, Qingdao Agricultural University, Qingdao 266109, China; 2 Qingdao Special Food Research Institute, Qingdao 266109, China; 3 Medical College, Shandong Xiehe University, Jinan 250000, China

**Keywords:** RS3, resistant starch, calcium alginate

## Abstract

In order to reduce the digestion rate of starch in human body and improve the content of slowly digestible starch (SDS) and resistant starch (RS), millimeter calcium alginate beads encapsulated with different proportions of recrystallized starch were constructed in this study. First, we prepared recrystallized starch (RS3) by debranching waxy corn starch and retrogradation, and then encapsulated RS3 in calcium alginate beads by the ionic gel method. The microstructure of the beads was observed by scanning electron microscope, and the gel texture properties, swelling properties, and in vitro digestibility of the beads were studied. The results showed that the beads after cooking still maintained high hardness and chewiness, and the swelling power and solubility of the beads were lower than that of native starch. Compared with native starch, the content of rapidly digestible starch (RDS) in beads decreased, while the content of SDS and RS increased. The sample with the highest content of RS is RS3_1_@Alginate_1_, whose content of RS is 70.10%, 52.11 times higher than that of waxy corn starch and 1.75 times higher than that of RS3. RS3 encapsulated in calcium alginate beads has a good encapsulation effect, and the content of SDS and RS is greatly increased. This study has important implications for reducing the digestion rate of starch and regulating the health of people with diabetes and obesity.

## 1. Introduction

With economic development and the improvement of living standards, diabetes, obesity and the metabolic problems associated with them have become important problems that affect human health. How to reduce energy intake while providing satiety is an urgent issue at present. Starch is an important component of daily energy and the main ingredient of staple food. According to the digestibility of starch in the human body, starch can be divided into rapidly digestible starch (RDS), slowly digestible starch (SDS), and resistant starch (RS) [1]. Resistant starch is the part of starch that cannot be digested in the small intestine but can be utilized in the colon by the gut flora [2,3]. Due to its nutritional properties, RS has been widely accepted as a novel dietary fiber [4]. Studies have shown that adding RS to a daily diet can effectively slow down the postprandial glycemic response, reduce total energy intake, and have a regulatory effect on body glycolipid metabolism [5].

In recent years, physically entrapped starch RS1 and retrograded starch RS3 have attracted the interest of researchers. Natural RS1 is ubiquitously expressed in coarsely ground cereals and legume cells. Due to the presence of a dense cell wall barrier on the outside of starch, it has the property of heat resistance [6].

RS3 is formed by recrystallization of starch after gelatinization in the cooling process. The preparation process is relatively easy to control, and there is no safety problem. It is the most promising type of RS. After gelatinization, the starch is fully hydrated and amylose escapes from the starch granules into the solution and randomly curls and winds to form a polymer. During the cooling process, the molecular chain of the polymer re-aggregates through hydrogen bonds to form a double helix structure [7]. The double helix structure forms a new crystal structure in the further retrogradation process. The retrogradation amylose is extremely difficult for enzymes to digest even if it is heated to 150 °C, and needs to be gelatinized completely at 154 °C~171 °C. The retrogradation amylopectin is easily digested and not heat-resistant [8,9,10]. Because RS3 is an important component of RS in the diet and is formed through food processing, it has great potential research value [10].

High-temperature treatment disrupts the RS3 double helix crystal structure. When the crystal structure is destroyed, the starch molecules in the free state are more susceptible to hydrolysis by digestive enzymes, so RS3 has the characteristics of heat intolerance. High-temperature treatment can damage the granule structure and degrade starch molecules [11], resulting in greatly reduced resistance, affecting the development of the RS industry and related low GI staple industries. Therefore, how to improve the heat resistance of RS is an urgent problem at present.

Inspired by native physically entrapped starch and retrograde starch, we found that debranched starch physically entrapped by calcium alginate has heat-resistant and digestion-resistant properties and plays an important role in the future development of functional dietary fibers. Sodium alginate is widely found in brown algae. The carboxyl group on the molecule can quickly cross-link with divalent metal ions, such as calcium ion, under mild conditions to form a dense gel. The gel has good biocompatibility, safety, and non-toxicity, and is widely used in the food field [12]. Calcium alginate can also be used for drug delivery. Man, J et al., selected calcium alginate microspheres with suitable sizes as the histone deacetylase inhibitor carrier (HDACi) and delivered the HDACi-loaded calcium alginate microspheres to mice with acute kidney injury by intravenous tail injection. The in vivo results showed that the HDACi-loaded calcium alginate microspheres could effectively reduce the renal regional inflammatory response and attenuate renal injury [13]. Alginate beads are a versatile system for various encapsulation techniques. Some studies have shown that sodium alginate and calcium ion could be crosslinked to embed starch to build microspheres, and it was found that the SDS was significantly lower than the native starch [14]. We constructed a new type of RS by physical entrapment of debranched starch by calcium alginate, combining the advantages of both physically entrapping resistant starch and retrograded resistant starch. We dissolved and mixed calcium alginate with debranched starch and dropped it into calcium chloride to form microspheres, which constituted an ideal outer shell of RS1, making the microspheres have excellent anti-swelling and anti-enzymatic hydrolysis abilities. The Ca^2+^ infiltrated in the microsphere core forms a dense structure with sodium alginate, and the debranched starch is embedded in the microsphere interior, so that the starch crystallization cannot be easily disrupted and has strong heat resistance [15]. Through the experiment we found that this new kind of RS microspheres has extremely desirable stability and digestion-resistance characteristics. After heating at 100 °C for 30 min, the RS content of the calcium-alginate-embedded debranched starch microspheres was promoted from 25.53% to 70.10%, which was 2.75-fold higher than that of RS3.

In this study, in order to reduce starch digestibility, we combined the preparation methods of RS1 and RS3. First, RS3 was prepared from debranched waxy corn starch, and then it was embedded in calcium alginate so that it could not be contacted by enzymes, thus achieving the effect of anti-digestion [10]. This study has great significance in reducing starch digestibility, maintaining residents’ health, preventing diabetes, obesity, and related metabolic problems.

## 2. Materials and Methods

### 2.1. Materials

Waxy maize starch from Shandong Fuyang Biotechnology Co., Ltd. (Dezhou, China). Pullulanase (E.C.3.2.1.41, 40 ASPU/mL) was obtained from Novozymes Investment Limited Company (Beijing, China). Medium viscosity sodium alginate and calcium chloride was obtained from Shenggong Biotechnology Co., Ltd. (Shanghai, China). Glucose oxidase–peroxidase assay kits (GOPOD) were purchased from Megazyme International Ireland Ltd. (Wicklow, Ireland). All other reagents came from Shenggong Biotechnology Co., Ltd. (Shanghai, China).

### 2.2. Preparation of RS3

RS3 was prepared according to the method of Sun et al. [12]. An amount of 10 g of waxy corn starch were weighed and added into 100 mL of phosphoric acid buffer solution with pH = 5.0 to prepare a waxy corn starch suspension (*m/v*) with a concentration of 10%. It was heated in a boiling water bath for 30 min and kept stirring to ensure the starch was fully gelatinized. It was taken down and cooled to 58 °C, and the pullulanase solution was added according to the amount of 30 ASPU/g starch, then the gelatinized starch solution was put into a thermostatic water bath at 58 °C for enzymolysis for 6 h, and the reaction was terminated after taking out and heating at 100 °C for 30 min. After centrifugation at 3000 rpm for 15 min to remove the precipitate, the supernatant was decanted and cooled to room temperature, and then stored in a refrigerator at 4 °C for 12 h for recrystallization. The self-assembled sample was washed several times at low temperature and then freeze-dried to obtain RS3.

### 2.3. Preparation of Calcium Alginate Encapsulated RS3 Gel Beads

**Figure 1:**
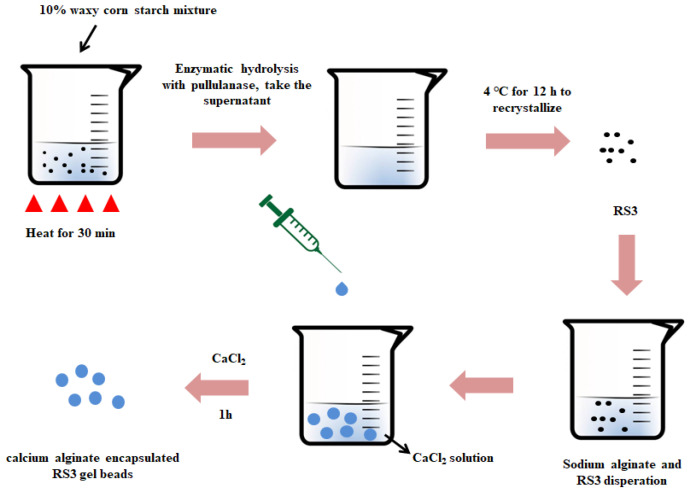
The process of manufacturing beads by the ionic crosslinking method.

As shown in Figure 1, the millimeter-sized beads are produced by the ionic cross-linking process. First, dissolve sodium alginate (2.0 g) in 100 mL distilled water. Then add 10 g of the previously prepared RS3 into the sodium alginate solution and the final concentration is 10% (*w*/*v*). They are collected in a filter and washed three times with distilled water. The prepared beads were dried in an oven at 45 °C for 24 h to obtain dry beads for subsequent physicochemical property and digestibility tests. The beads were prepared without any pH adjustment. There have been some relevant reports on the preparation of calcium alginate beads, but no research on the embedding of debranched starch by calcium alginate beads has been reported yet.

### 2.4. Morphological Characteristics of Beads

The morphological characteristics of gel beads were observed by digital camera, ordinary optical microscope, and scanning electron microscope. When observed by scanning electron microscope, the samples were observed after spraying with gold.

### 2.5. Texture Profile Analysis of Beads

Texture profile is one of the most important indexes to evaluate food quality. TPA testing was conducted using a texture analyzer (TMS-PRO, FTC Corporation of the United States). The flat-bottom cylindrical probe P 36R was used to test the fresh beads and the cooked beads. Test conditions: The probe descending speed is 1.0 m/s, the return speed is 1.0 m/s, and the deformation force is 50%. Ten beads were selected for a single test to improve test accuracy. Each sample was tested three times and then averaged [14,16].

### 2.6. Swelling Behavior Measurements of the Beads

The native starch and beads were mixed into a 1.0% (*w*/*w*) solution, which was continuously heated at 55 °C, 65 °C, 75 °C, 85 °C, and 95 °C for 30 min, respectively, and gently stirred with a glass rod every 5 min. The starch samples were then centrifuged at 3000 rpm for 15 min, and the supernatant was placed in an oven to dry at 105 °C to a constant weight and weighed to obtain the amount of dissolved starch (A). The swelling power and solubility were calculated in combination with the precipitate slurry weight (B) in the centrifuge tube.

Solubility refers to the percentage of dissolved mass of starch molecules at a certain temperature. Starch granules swelled in hot water, and a portion of starch was dissolved in water, and the solubility was calculated at the conditions of 55 °C, 65 °C, 75 °C, 85 °C, and 95 °C, respectively:

%SOL = A/S × 100%(1)

Swelling power refers to the mass number after water absorption at a certain temperature per gram of dry starch. Swelling power = (mass of precipitate × 100)/dry basis mass of starch × (100% − soluble carbohydrates as a percentage of dry-based starch), i.e.:

SP = (B × 100)/S(1 − %SOL)(2)

### 2.7. Determination of Total Starch Content

A sample of 2 g was first weighed, ground and passed through a 40-mesh sieve. The fat in the sample was washed off three times with 30 mL of diethyl ether, and the residue was washed again three times with 150 mL of 85% ethanol to remove soluble sugars. The residue was dissolved with 100 mL of water, and 30 mL of 6 mol/L hydrochloric acid was added and refluxed for 2 h before cooling with flowing water. Methyl red indicator solvent was added, and the sample hydrolysate was adjusted for neutralization by 40% NaOH solution and 6 mol/L hydrochloric acid. Then 20 mL of 20% neutral lead acetate solution was added to precipitate proteins, pectin, and other impurities. Another 20 mL of 10% NaSO_4_ solution was added to remove excessive lead. The amount of total glucose in the solution was determined using GOPOD, and the total starch content was calculated using the following equation [17]:

(3) $$TS(\%)=\frac{\Delta A\times F\times FV\times 0.9}{W}$$

(4) $$F=\frac{100(ug\;of\;D-glucose)}{absorbance\;for\;100\;ug\;of\;glucose}$$

Δ*A* is the absorbance of the reagent blank, *FV* is the final volume of the analyte (mL), and *W* is the weight (mg) of the sample to be analyzed.

### 2.8. In Vitro Digestibility of Beads

Each 200 mg sample was weighed (based on dry starch), 18 mL sodium acetate buffer solution was added (pH 5.20) in a 100 mL conical flask, and the mixture was heated for 30 min at 100 °C. The mixture was cooled to 37 °C, and 2 mL of newly prepared mixed enzyme solution (pancreatin and amyloglucosidase) was added. The process of enzymatic hydrolysis was carried out in a 37 °C constant temperature shaker. At the digestion time of 0, 20, 60, 90, 120, and 180 min, 0.1 mL reaction solution was mixed with 0.9 mL 66% ethanol solution to terminate the reaction. After centrifugation at 4000 rpm/min for 10 min, 0.1 mL of the supernatant was taken and the content of glucose in the supernatant was measured using the GOPOD kit. The contents of RDS, SDS and RS are calculated according to the following formulas [18,19]:

%RDS = (G20 − G0) × 0.9 × 100/S(5)

%SDS = (G120 − G20) × 0.9 × 100/S(6)

%RS = [TS − (RDS + SDS)] × 100/S(7)

The conversion coefficient between glucose content and starch is 0.9. G0, G20, and G120 represent the glucose content produced after enzymatic hydrolysis of the sample at 0, 20, and 120 min, while S represents the total starch content of the sample (calculated on a dry basis).

In order to simulate the digestion of the human gastrointestinal tract, a first-order kinetic equation was used to fit the digestion curve of starch. The fitting equation is as follows:

C = 1 − e^−kt^ (8)

C(%) is the proportion of digested starch to total starch at time t (min) and K is the digestion rate coefficient (min^−1^). The remaining undigested starch after time t (min) is 1 − C [20].

### 2.9. Statistical Analysis

All indexes were performed in three independent repeated tests, and the results were expressed as mean ± standard deviation. SPSS 26 software was used to process the test data, and the significance of difference was determined by Duncan’s method, and the significant difference was defined as *p* < 0.05. Plotting was performed using origin Pro 8.5.

## 3. Results and Discussion

### 3.1. Morphology of RS3

**Figure 2:**
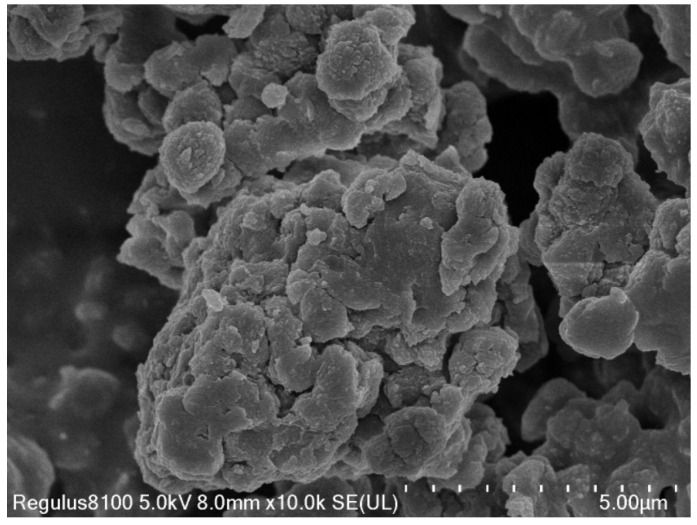
SEM image of RS3.

The prepared RS3 was observed by SEM (Figure 2), and granular or rod-shaped starch spherulites could be seen. Among them, the particles are close to microsphere, and some particles are nanospheres, but the starch spherulites almost all gather together to form larger particles. The results showed that waxy corn starch was crystallized after pullulanase debranching and retrogradation. The particle surface formed by rearrangement is relatively dense and has no holes, which is not conducive to the adsorption of enzymes, so the hydrolysis rate is relatively slow. Hung et al. [21] found that the intake of recrystallized RS3 can reduce the blood glucose release level of mice. A randomized, single-blind cross-over study by M. H. Alhussain et al., examined the effects of RS3 on postprandial glucose and insulin responses and appetite in young healthy men. Studies have found that consuming RS3 meals is associated with greater satiety and lower appetite, which may lead to long-term weight management [22]. In view of the beneficial effect of RS3 on health, we prepared a composite type of resistant starch by using the prepared RS3 as raw material.

### 3.2. Morphology of Calcium Alginate Encapsulated RS3 Beads

**Figure 3:**
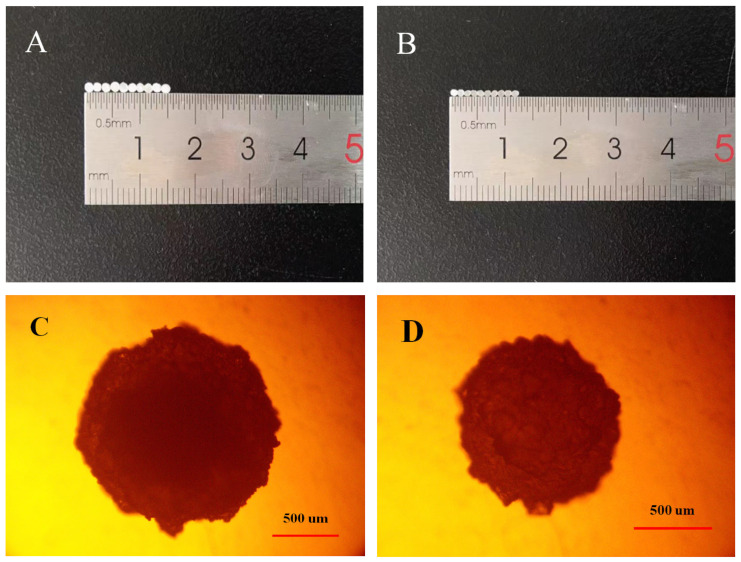
Photos of dried gel beads (**A**,**C**) RS3_5_@Alginate_1_ (**B**,**D**) RS3_1_@Alginate_1._

**Figure 4:**
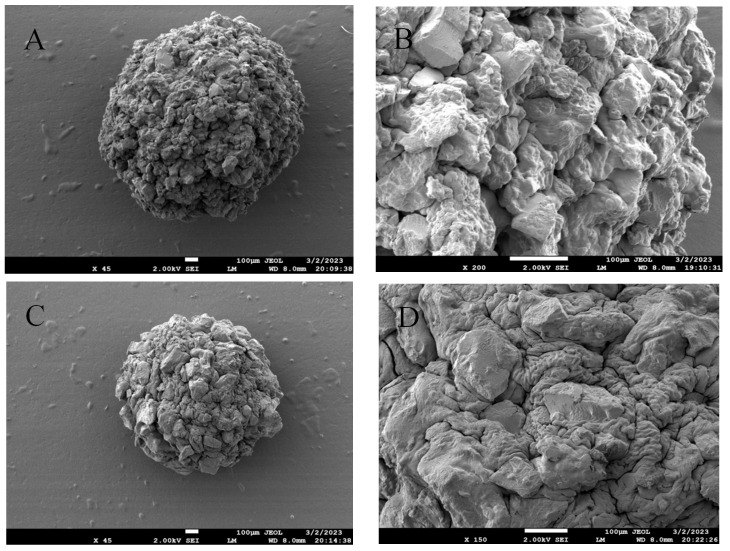
SEM photo of gel beads (**A**,**B**) RS3_5_@Alginate_1_ (**C**,**D**) RS3_1_@Alginate_1._

The diameter of the dried gel beads is about 1.2~1.5 mm (Figure 3). The beads are spherical, and the surface is slightly rough. When the ratio of RS3 to calcium alginate is 1:1, the dried calcium alginate gel beads produced are translucent with a small diameter of about 1.2 mm. When the ratio of RS3 to calcium alginate is 5:1, the dried calcium alginate gel beads produced are a slightly transparent white with a large diameter of about 1.5 mm. The SEM photo of the dried gel beads is shown in Figure 4. A layer of calcium alginate was found on the surface of the starch, which is wrapped around the surface of the starch granules. This is because sodium alginate and calcium ions can form gel beads to wrap starch [23]. In addition, Feltre, G et al., prepared natural maize starch microspheres using sodium alginate suspension dropwise into calcium chloride solution. It was found that corn starch alginate microspheres had lower water uptake, higher gelatinization temperature, and a wider range of gelatinization temperatures than native corn starch [24].

### 3.3. Gel Texture Properties

The texture properties of fresh calcium alginate gel beads are shown in Table 1. For the samples of gel beads with different RS3 concentrations, the hardness of gel beads with higher starch concentration is higher. It indicates that high RS3 addition may form a strong starch calcium alginate gel. At the same time, gel beads with higher starch concentration have higher chewiness. For instance, the chewiness of RS3_5_@Alginate_1_ is 234.75 ± 33.43, which is significantly higher than RS3_1_@Alginate_1_ and calcium alginate beads. The adhesiveness and springiness of fresh gel beads are relatively low.

In addition, the texture properties of dried gel beads after 30 min cooking at 100 °C were also measured, as shown in Table 2. The hardness of gel beads increased significantly after cooking, which may be due to starch gelatinization. Compared with fresh samples, the chewiness of cooked beads is also improved, which may be because gelatinized starch increases the chewiness of cooked samples. There is no significant change in the values of adhesiveness and springiness.

### 3.4. Swelling Power (SP) and Solubility (S)

When heated, the starch granules absorb water and expand, and some water-soluble components are dissolved in water. Similarly, the starch in the sample expands and dissolves after the beads absorb water. SP and S values of various beads at different temperatures are shown in Table 3. At the same temperature, the SP and S values of gel bead samples were reduced compared to waxy maize starch and RS3.

For example, at 95 °C, the swelling power of RS3_1_@Alginate_1_ is 9.94 ± 0.14 g/g, and its solubility is 7.42 ± 0.21%, which is 64.2% and 51.3% lower than RS3, respectively, 77.0% and 61.5% lower than waxy corn starch. This result indicated that the swelling of RS3 was inhibited by calcium alginate gel structure. Moreover, the higher the addition of calcium alginate, the lower the swelling power and solubility of the gel beads. This is because, in general, the stronger the crosslinked network of calcium alginate, the higher the degree of inhibition. This result is similar to that of Feltre, G. et al., who found lower water uptake by corn starch alginate microspheres than native corn starch. Lower water uptake is associated with lower swelling and solubility [24].

### 3.5. Determination of Total Starch Content

The total starch content of RS3_5_@Alginate_1_ is 80.62 ± 0.46%, and the total starch content of RS3_1_@Alginate_1_ is 47.31 ± 0.35% (Table 4). The total starch content of the prepared beads is very close to the proportion of starch added during preparation, indicating that the prepared calcium-alginate-encapsulated RS3 gel beads have a good encapsulation effect. Najafi-Soulari, S. et al., used calcium alginate hydrogel beads to encapsulate lemon balsam extract. It was found that calcium alginate had a high encapsulation efficiency, and there was no obvious change in the antioxidant activity of the extract of vetiver after encapsulation. Therefore, alginate was found to be a suitable encapsulation material for natural antioxidants [25].

### 3.6. Determination of In Vitro Digestibility

After 30 min of boiling water treatment, the measured values of RDS, SDS and RS of native starch and the beads are shown in Table 5. The RDS content of RS3_5_@Alginate_1_ is 31.87%, which is 65.89% lower than that of waxy corn starch and 52.95% lower than that of RS3. Moreover, the RDS content of RS3_1_@Alginate_1_ is 20.85%, which is 77.68% lower than that of waxy corn starch and 69.22% lower than that of RS3. The results showed that calcium-alginate-embedded RS3 had a good effect on reducing starch digestibility. In addition, with the increase in calcium alginate added, the content of SDS and RS in the sample also increased. For example, the sample with the highest RS content is RS3_1_@Alginate_1_ and its RS content reaches 70.10%, which is 52.11 times higher than that of waxy corn starch and 1.75 times higher than that of RS3. This may be because the calcium alginate network prevents the contact between starch and amylase, thus inhibiting the hydrolysis of starch. Similarly, Cui et al., reported that corn starch in calcium alginate microspheres can reduce the content of RDS in corn starch and the content of RS reached 18.3% [26]. Qin et al., reported that corn starch, pea starch, and potato starch encapsulated in chitosan capsules can reduce the digestibility of native starch, with RS content reaching 13.35%, 20.12%, and 14.58% [27]. However, when we use calcium alginate to embed debranched starch, the content of RS can reach 70.10%, which is much higher than that of starch gel beads obtained by traditional methods. Foods with high RS content can delay the postprandial blood glucose response of obese and diabetes people, and play an important role in the regulation of glucose homeostasis.

### 3.7. Hydrolysis Curve

**Figure 5:**
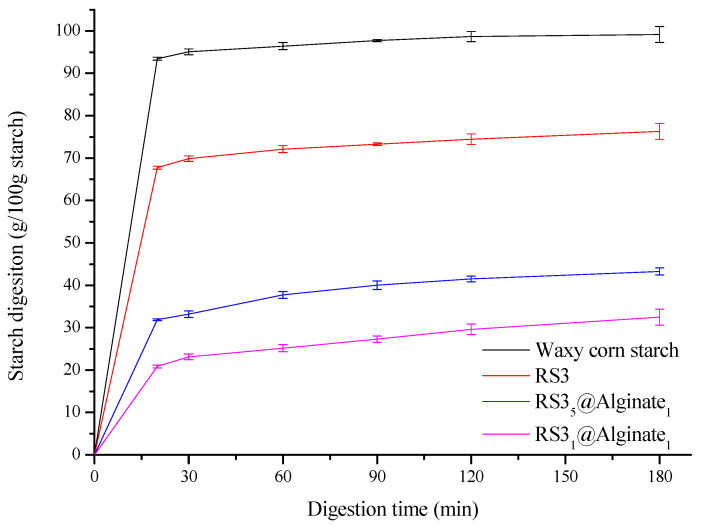
Hydrolysis curve of native starch and different samples.

The hydrolysis curve of native starch and sample is shown in Figure 5. When the ratio of calcium alginate to RS3 is 1:5, the digestibility of starch after 20 min of amylase digestion is 31.87%, while when the ratio of calcium alginate to RS3 is 1:1, the digestibility of starch is 20.85%, while the digestibility of RS3 after 20 min of digestion is 67.73%, and the digestibility of waxy corn starch after 20 min of digestion is 93.43%. RS3_1_@Alginate_1_ has the lowest digestion rate and final digestion ratio, while waxy corn starch has the highest digestion rate and final digestion ratio. In general, the digestion curve of calcium alginate beads wrapped with RS3 is relatively flat, while that of raw starch and RS3 beads not wrapped with calcium alginate is relatively steep. This is because the coating of calcium alginate makes it difficult for starch to have contact with enzymes, thus slowing down the glucose production of starch.

**Figure 6:**
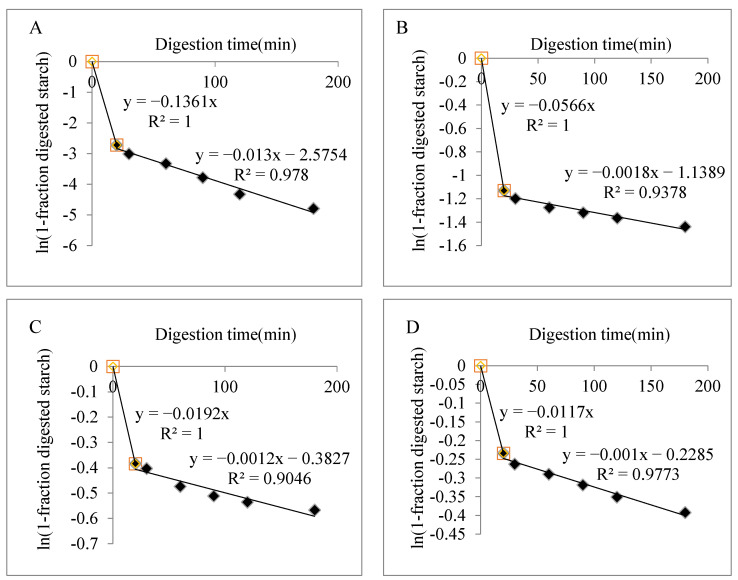
Fit of first-order kinetics of native starch and bead samples. (**A**) Waxy corn starch (**B**) RS3 (**C**) RS3_5_@Alginate_1_ (**D**) RS3_1_@Alginate_1._

According to the hydrolysis curve in Figure 5, the digestion rate of starch gel beads is fast in 0–20 min and tends to be slow in 20–120 min, so we use two-stage kinetics to fit the hydrolysis curve. As can be seen from Figure 6, the first-order kinetic fitting results for both native starch and beads exhibited two-stage kinetics. The k values of all samples at 0–20 min were larger than those at 20–180 min, that is, k1 > k2. This is because the digestion rate of starch at 0–20 min was faster than that at 20–180 min. In addition, k1 and k2 decreased with the increase in the calcium alginate ratio. This shows that the higher the amount of calcium alginate added, the lower the digestibility of the sample within 0–180 min. This is because the calcium alginate film on the surface of the beads prevents the amylase from contacting the starch.

## 4. Conclusions

We propose a new strategy to improve the content of SDS and RS. First, recrystallized starch (RS3) was prepared, and then a calcium alginate layer was formed on the surface of RS3 to make calcium-alginate-encapsulated RS3 beads. The results showed that the beads after cooking still maintained high hardness and chewiness, and the swelling power and solubility of the beads are lower than those of starch. Compared with native starch, the content of RDS in the beads decreased, while the content of SDS and RS increased. The sample with the highest content of RS is RS3_1_@Alginate_1_, which content of RS is 70.10%, 52.11 times higher than that of waxy corn starch and 1.75 times higher than that of RS3. During digestion, calcium alginate acts as a physical barrier to prevent amylase from digesting starch. The results showed that the calcium alginate film was coated on the surface of the starch, providing protection to the starch, preventing amylase from penetrating, and slowing down starch digestion. This result combines the advantages of RS1 and RS3, and overcomes the shortcomings of traditional single methods of preparing resistant starch. The prepared calcium-alginate-embedded RS3 beads not only have high resistant starch content, but also have good thermal stability. In addition, the preparation process did not introduce additional chemical groups, and the prepared resistant starch has green and safe properties, making it easy for the public to accept. This study can increase the content of slow digestible starch and resistant starch, and has good application prospects, which is of great significance for maintaining the health of patients with chronic diseases related to blood sugar.

## Figures and Tables

**Table 1:** Texture properties of fresh beads.

Sample	Hardness (g)	Adhesiveness (g.sec)	Chewiness	Springiness
Calcium alginate beads	475.37 ± 4.29 ^c^	−4.29 ± 0.11 ^a^	182.36 ± 6.27 ^c^	0.72 ± 0.03 ^a^
RS3_1_@Alginate_1_	509.43 ± 3.34 ^b^	−1.76 ± 0.04 ^b^	216.44 ± 11.73 ^b^	0.76 ± 0.01 ^a^
RS3_5_@Alginate_1_	582.34 ± 16.46 ^a^	−0.46 ± 0.02 ^c^	234.75 ± 33.43 ^a^	0.82 ± 0.01 ^b^

Values mean ± SD indicate the replicates of three experiments; values with different letters (a, b, c) are significantly different (*p* < 0.05).

**Table 2:** Texture properties of beads after cooking.

Sample	Hardness (g)	Adhesiveness (g.sec)	Chewiness	Springiness
Calcium alginate beads	2764.26 ± 163.54 ^a^	−0.05 ± 0.01 ^c^	1132.24 ± 4.65 ^b^	0.78 ± 0.01 ^a^
RS3_1_@Alginate_1_	2237.46 ± 127.35 ^b^	−1.34 ± 0.03 ^b^	1056.61 ± 17.34 ^a^	0.83 ± 0.01 ^a^
RS3_5_@Alginate_1_	1836.31 ± 27.48 ^c^	−3.24 ± 0.03 ^a^	1046.35 ± 41.27 ^a^	0.89 ± 0.01 ^a^

Values mean ± SD indicate the replicates of three experiments; values with different letters (a, b, c) are significantly different (*p* < 0.05).

**Table 3:** Swelling characteristics of beads.

Temperature (°C)	Swelling Power (g/g)					Solubility (%)				
	55 °C	65 °C	75 °C	85 °C	95 °C	55 °C	65 °C	75 °C	85 °C	95 °C
Waxy corn starch	10.35 ± 0.12 ^a^	12.54 ± 0.14 ^a^	34.48 ± 0.27 ^a^	35.57 ± 0.37 ^a^	43.22 ± 0.37 ^a^	4.4 ± 0.02 ^a^	6.63 ± 0.07 ^a^	7.36 ± 0.18 ^a^	9.68 ± 0.19 ^a^	19.26 ± 0.26 ^a^
RS3	4.27 ± 0.21 ^b^	8.65 ± 0.23 ^b^	18.87 ± 0.29 ^b^	23.83 ± 0.56 ^b^	38.37 ± 0.42 ^b^	3.2 ± 0.02 ^b^	5.12 ± 0.03 ^b^	6.14 ± 0.21 ^b^	8.31 ± 0.13 ^b^	15.24 ± 0.28 ^b^
RS3_5_@Alginate_1_	3.38 ± 0.02 ^c^	5.21 ± 0.06 ^c^	9.36 ± 0.14 ^c^	11.57 ± 0.13 ^c^	13.21 ± 0.24 ^c^	2.31 ± 0.03 ^c^	4.12 ± 0.04 ^c^	5.24 ± 0.16 ^c^	7.42 ± 0.13 ^c^	9.56 ± 0.14 ^c^
RS3_1_@Alginate_1_	2.63 ± 0.13 ^d^	3.74 ± 0.13 ^d^	5.73 ± 0.04 ^d^	8.53 ± 0.17 ^d^	9.94 ± 0.14 ^d^	1.24 ± 0.02 ^d^	2.68 ± 0.04 ^d^	3.73 ± 0.13 ^d^	5.52 ± 0.14 ^d^	7.42 ± 0.21 ^d^

Values mean ± SD indicate the replicates of three experiments; values with different letters (a, b, c, d) are significantly different (*p* < 0.05).

**Table 4:** Ingredients list of native starch and samples.

Samples	TS(%)	Concentration of Sodium Alginate (% of 100 mL Water Solution)	Concentration of Calcium Chloride (% of 100 mL Water Solution)	Debranched Starch (% of 100 mL Water Solution)
Waxy corn starch	98.57 ± 0.34 ^a^	/	/	/
RS3	97.48 ± 0.27 ^a^	/	/	/
RS3_5_@Alginate_1_	80.62 ± 0.46 ^b^	2	1	10
RS3_1_@Alginate_1_	47.31 ± 0.35 ^c^	2	1	2

Values mean ± SD indicate the replicates of three experiments; values with different letters (a, b, c) are significantly different (*p* < 0.05).

**Table 5:** Contents of RDS, SDS, and RS of cooked native starch and beads.

Samples	RDS (%)	SDS (%)	RS (%)
Waxy corn starch	93.43 ± 0.32 ^a^	5.25 ± 0.15 ^d^	1.32 ± 0.17 ^d^
RS3	67.73 ± 0.24 ^b^	6.74 ± 0.16 ^c^	25.53 ± 0.39 ^c^
RS3_5_@Alginate_1_	31.87 ± 0.22 ^c^	7.62 ± 0.17 ^b^	60.51 ± 0.43 ^b^
RS3_1_@Alginate_1_	20.85 ± 0.34 ^d^	9.05 ± 0.33 ^a^	70.10 ± 0.64 ^a^

Values mean ± SD indicate the replicates of three experiments; values with different letters (a, b, c, d) are significantly different (*p* < 0.05).

## Data Availability

Data is contained within the article.

## References

[1] Englyst H.N., Kingman S.M., Cummings J.H. (1992). Classification and measurement of nutritionally important starch fractions. Eur. J. Clin. Nutr..

[2] Wang M.W., Chen X.Y., Zhou L.Y., Li Y., Yang J., Ji N., Xiong L., Sun Q.J. (2022). Prebiotic effects of resistant starch nanoparticles on growth and proliferation of the probiotic Lactiplantibacillus plantarum subsp. plantarum. LWT-Food Sci. Technol..

[3] Peter A.D., Alain S. (2021). Resistant starch, microbiome, and precision modulation. Gut. Microbes.

[4] Miao T.T., Xiong K., Ji N., Xiong L., Sun C.R., Li X.J., Ma A.G., Sun Q.J. (2020). Resistant starch nanoparticles prepared from debranched starch by medium-temperature recrystallization. Int. J. Biol. Macromol..

[5] Wali J.A., Milner A.J., Luk A.W., Pulpitel T.J., Dodgson T., Facey H.J., Wahl D., Kebede M.A., Senior A.M., Sullivan M.A. (2021). Impact of dietary carbohydrate type and protein–carbohydrate interaction on metabolic health. Nat. Metab..

[6] Kraithonga S., Wang S.K., Junejo S.A., Fu X., Theppawong A., Zhang B., Huang Q. (2022). Type 1 resistant starch: Nutritional properties and industry applications. Food Hydrocoll..

[7] Hsein-Chih H.W., Anatole S. (1978). The double-helical molecular structure of crystalline α-amylose. Carbohydr. Res..

[8] Ring S.G., Gee J.M., Whittam M., Orford P., Johnson I.T. (1988). Resistant starch: Its chemical form in food stuffs and effectson digestibility in vitro. Food Chem..

[9] Eerlingen R.C., Broeck V.D., Delcour J.A., Slade L., Levine H. (1994). Enzyme resistant starch VI: Influence of sugars on resistant starch formation. Cereal Chem..

[10] Frost G., Leeds A.A., Dore C.J., Madeiros S., Brading S., Dornhorst A. (1999). Glycemic index as a determinant of serum HDL-cholesterol concentration. Lancet.

[11] Liu S., Reimer M., Ai Y. (2020). Yongfeng. In vitro digestibility of different types of resistant starches under high-temperature cooking conditions. Food Hydrocoll..

[12] Sun Q.J., Li G., Dai L., Ji N., Xiong L. (2014). Green preparation and characterisation of waxy maize starch nanoparticles through enzymolysis and recrystallisation. Food Chem..

[13] Man J., Wang X.J., Li J.Y., Cui X.Y., Hua Z.S., Li J.F., Mao Z.B., Zhang S.G. (2022). Intravenous Calcium Alginate Microspheres as Drug Delivery Vehicles in Acute Kidney Injury Treatment. Micromachines.

[14] Lozano-Vazquez G., Lobato-Calleros C., Escalona-Buendia H., Chavez G., AlvarezRamirez J., Vernon-Carter E.J. (2015). Effect of the weight ratio of alginatemodified tapioca starch on the physicochemical properties and release kinetics of chlorogenic acid containing beads. Food Hydrocoll..

[15] Wang P., Luo Z.G., Xiao Z.G. (2021). Preparation, physicochemical characterization and in vitro release behavior of resveratrol-loaded oxidized gellan gum/resistant starch hydrogel beads. Carbohydr. Polym..

[16] Li W., Cao F., Fan J., Ouyang S., Luo Q., Zheng J., Zhang G. (2014). Physically modified common buckwheat starch and their physicochemical and structural properties. Food Hydrocoll..

[17] Giuberti G., Gallo A. (2017). Reducing the glycaemic index and increasing the slowly digestible starch content in gluten-free cereal-based foods: A review. Int. J. Food Sci. Technol..

[18] Cui C., Li M., Ji N., Qin Y., Shi R., Qiao Y., Xiong L., Dai L., Sun Q. (2022). Calcium alginate/curdlan/corn starch@calcium alginate macrocapsules for slowly digestible and resistant starch. Carbohydr. Polym..

[19] Borczak B., Sikora E., Sikora M., Kapusta-Duch J., Rosell C.M. (2015). Starch digestibility index and antioxidative properties of partially baked wheat-flour bakery with an addition of dietary fibre. Starch Starke.

[20] Dhital S., Shrestha A.K., Gidley M.J. (2010). Effect of cryo-milling on starches: Functionality and digestibility. Food Hydrocoll..

[21] Hung P.V., Binh V.T., Nhi P.H.Y., Phi N.T.L. (2020). Effect of heat-moisture treatment of unpolished red rice on its starch properties and in vitro digestibility. Int. J. Biol. Macromol..

[22] Alhussain M.H., Almousa A., Alhowikan A. (2021). Impact of resistant starch type-3 on glucose metabolism and appetite in healthy males. Proc. Nutr. Soc..

[23] Zafeiri I., Beri A., Linter B., Norton I. (2021). Mechanical properties of starch-filled alginate gel particles. Carbohydr. Polym..

[24] Feltre G., Silva C.A., Lima G.B., Menegalli F.C., Dacanal G.C. (2018). Production of Thermal-Resistant Cornstarch-Alginate Beads by Dripping Agglomeration. Int. J. Food Eng..

[25] Najafi-Soulari S., Shekarchizadeh H., Kadivar M. (2016). Encapsulation optimization of lemon balm antioxidants in calcium alginate hydrogels. J. Biomater. Sci. Polym. Ed..

[26] Cui C.L., Jiang H., Guan M.H., Ji N., Xiong L., Sun Q.J. (2022). Characterization and in vitro digestibility of potato starch encapsulated in calcium alginate beads. Food Hydrocoll..

[27] Qin K.L., Sun D.Y., Wang C.F., Ji N., Dai L., Qin Y., Xiong L., Wang T., Sun Q.J. (2023). Properties and in vitro digestibility of starch encapsulated in chitosan-sodium phytate capsules. Food Hydrocoll..

